# Virulence and mutations analysis based on the whole genome of a Brazilian *Corynebacterium diphtheriae* strain isolated from a cutaneous infection

**DOI:** 10.3389/fmicb.2025.1579154

**Published:** 2025-10-15

**Authors:** Max Roberto Batista Araújo, Louisy Sanches dos Santos, Fernanda Diniz Prates, Hugo Felix Perini, Jailan Sousa Silva, Juliana Nunes Ramos, Sérgio Bokermann, Cláudio Tavares Sacchi, Ana Luíza de Mattos Guaraldi, Karoline Rodrigues Campos, Tayná do Carmo Sant’Anna Cardoso, Diogo Luiz de Carvalho Castro, Marcos Andrade Silva, Mireille Ângela Bernardes Sousa, Verônica Viana Vieira, Marlon Benedito Nascimento Santos, Carlos Henrique Camargo, Bruno Silva Andrade, Marcos Vinicius da Silva, Lincoln de Oliveira Sant’Anna, Marcus Vinícius Canário Viana, Vasco Azevedo

**Affiliations:** ^1^Operational Technical Nucleus, Microbiology, Hermes Pardini Institute (Fleury Group), Vespasiano, Brazil; ^2^Department of Genetics, Ecology and Evolution, Federal University of Minas Gerais, Belo Horizonte, Brazil; ^3^Laboratory of Diphtheria and Corynebacteria of Clinical Relevance, Department of Microbiology, Immunology and Parasitology, Rio de Janeiro State University, Rio de Janeiro, Brazil; ^4^Institute of Biological and Natural Sciences, Federal University of Triângulo Mineiro, Uberaba, Brazil; ^5^Center of Bacteriology, Adolfo Lutz Institute, Secretary of Health of the State of São Paulo, São Paulo, Brazil; ^6^Strategic Laboratory, Adolfo Lutz Institute, Secretary of Health of the State of São Paulo, São Paulo, Brazil; ^7^Interdisciplinary Laboratory of Medical Research, Oswaldo Cruz Institute, Oswaldo Cruz Foundation, Rio de Janeiro, Brazil; ^8^Laboratory of Bioinformatics and Computational Chemistry, State University of Southwest Bahia, Jequié, Brazil

**Keywords:** *Corynebacterium diphtheriae* complex, non-toxigenic, virulence factors, resistance genes, CRISPR-Cas system

## Abstract

*Corynebacterium diphtheriae* is the main etiological agent of diphtheria, a potentially fatal disease whose most severe signs and symptoms result from the action of an exotoxin, the diphtheria toxin (DT). Although non-toxigenic *C. diphtheriae* strains have been associated with several diseases, including cutaneous infections and endocarditis, they are not monitored in many countries, and their mechanisms of virulence and antimicrobial resistance remain underexplored. Therefore, this study aimed to provide a comprehensive characterization -through genomic, *in vitro*, and *in vivo* analyses - of a non-toxigenic *C. diphtheriae* strain (46855) isolated from a leg lesion, highlighting its pathogenic potential and resistance profile. The isolate was assigned to a novel sequence type (ST-925) and was found to be resistant to tetracycline and rifampin. Multiple antimicrobial resistance genes were predicted in the genome, such as tet(33), rbpA, and rpoB2, in addition to mutations in the rpoB gene. A diverse set of virulence-associated genes related to adhesion, iron uptake systems, gene regulation, and post-translational modification was also identified. The isolate was able to form biofilm *in vitro* and exhibited strong virulence in *Galleria mellonella* larvae and A549 human pneumocyte cells. Finally, the structural analysis of the rpoB gene, carried out for the first time in this study, linked the observed mutations to rifampin resistance in *C. diphtheriae*. In summary, the data revealed that *C. diphtheriae* 46855, although non-toxigenic, harbors multiple genes associated with antimicrobial resistance and virulence, emphasizing the need for greater surveillance and functional studies on non-toxigenic strains.

## 1 Introduction

The genus *Corynebacterium* currently includes 167 valid species (Parte et al., 2020), the most well-known of which is *Corynebacterium diphtheriae*, the leading causative agent of diphtheria, a potentially fatal disease that affects the respiratory tract and, occasionally, the skin (Prygiel et al., 2022). Diphtheria toxin (DT) is the main virulence factor of *C. diphtheriae*, encoded by the *tox* gene carried by corynebacteriophages that can lysogenize species of *Corynebacterium*, leading to the conversion of a non-toxigenic isolate into a toxigenic one (Sangal and Hoskisson, 2020).

Although vaccination with the diphtheria toxoid has drastically reduced the number of diphtheria cases worldwide, the disease is still reported. In recent years, outbreaks have occurred in countries such as India, Algeria, Guinea, Niger, and Nigeria (Africa Centres for Disease Control and Prevention, 2023; Sharma et al., 2022). Diphtheria cases, mostly in the cutaneous form, have also been reported in European countries; only between 2022 and 2023, 281 cases were documented, with the majority reported by Germany and Belgium (European Centre for Disease Prevention and Control, 2023). The persistence of diphtheria cases reports is primarily attributed to inadequate vaccine coverage. However, given that variations in the *tox* gene have been detected among circulating isolates (Will et al., 2021), the possibility of the emergence of structurally distinct DT-producing strains, potentially capable of escaping vaccine-induced immunity, should also be considered.

Non-toxigenic strains of *C. diphtheriae* have also been associated with human infections, including invasive diseases and cutaneous infections (Batista Araújo et al., 2021; Gomes et al., 2009; Lowe et al., 2011; Peixoto et al., 2020; Ramos et al., 2024; Sharma et al., 2019). It is known that both toxigenic and non-toxigenic *C. diphtheriae* may be found colonizing pre-existing skin lesions, such as surgical wounds, burns, and insect bites, mainly on the legs, feet, and hands. These infected lesions act as reservoirs of this pathogen that can contaminate the environment and induce human infections in contact more efficiently than pharyngeal infections, contributing to the emergence of outbreaks and epidemics of diphtheria in vulnerable populations (Chêne et al., 2024).

Whole-genome sequencing (WGS) has been widely adopted in global surveillance initiatives to track bacterial pathogens. Recent efforts, including the World Health Organization’s Global Genomic Surveillance Strategy for Pathogens with Pandemic and Epidemic Potential (2022–2032) (World Health Organization, 2022), underscore the central role of WGS in public health surveillance and response. In this sense, genomic sequencing has been employed for the identification, molecular typing, and characterization of *C. diphtheriae* strains (Chorlton et al., 2020; Meinel et al., 2016; Ramos et al., 2024; Seth-Smith and Egli, 2019; Setiawaty et al., 2022; Will et al., 2021; Wołkowicz et al., 2023), providing insights into the genomic diversity and evolution of this species. However, unlike toxigenic strains, non-toxigenic *C. diphtheriae* remain under-monitored in many regions, particularly in developing countries, resulting in limited data on their epidemiology and clinical impact. Moreover, their mechanisms of antimicrobial resistance and virulence are still poorly understood, raising concerns about their potential role as reservoirs of resistance/virulence genes and as contributors to the overall disease burden.

In the present study we provided an integrative characterization, combining genomic, *in vitro*, and *in vivo* analyses, of a non-toxigenic but virulent strain of this species isolated from a leg lesion of a immunized adolescent residing in a state of Brazil. Besides, we provided, for the first time, a structural analysis of mutations carried by this isolate that can be linked to rifampin resistance.

## 2 Materials and methods

### 2.1 Origin of bacterial strains

A 16-years-old Brazilian male patient, a resident of the rural area of a municipality in the State of Ceará, with no previous comorbidity diagnosis and difficult access to health systems, presented ulcerated lesions on his legs. After presenting it to the general practitioner, a swab from the lesion’s secretion was collected and sent for laboratory analysis.

A culture of swab was performed on 5% sheep’s blood agar (bioMérieux^®^, Brazil) by incubation at 37 °C for 48 h. For identification of isolates, MALDI-TOF MS (Matrix Assisted Laser Desorption Ionization Time-of-Flight Mass Spectrometry) analysis in the semi-automated system VITEK^®^ MS (bioMérieux^®^, France) was used. A bacterial spot of 1–3 colonies was placed on the target slide with the addition of 1 μL α-cyano-4-hydroxycyanic acid matrix - VITEK MS-CHCA (bioMérieux^®^, Brazil). After drying and crystallizing the matrix and sample, the slide was introduced into the VITEK^®^ MS system to acquire the protein mass spectra, mainly composed of ribosomal protein. The obtained mass spectra were compared with the MYLA^®^ software version 4.7.1 database (bioMérieux^®^, France), recommended for clinical use or Ruo SARAMIS^®^ software version 4.16 (research use only) - Spectral Archive and Microbial Identification System (bioMérieux^®^, France), that allow the identification of the genus and species.

In addition, the bacterial isolate was phenotypically characterized using the Analytical Profile Index (API)^®^ Coryne System (bioMérieux^®^, France).

### 2.2 Antimicrobial susceptibility *in vitro*

The antimicrobial profile of the clinical isolate was determined using the disk diffusion method according to the guideline provided by the Brazilian Committee on Antimicrobial Susceptibility Testing (BrCAST) (2020, 2024). Bacterial suspension of the isolate was prepared in saline with the turbidity equivalent to the 0.5 McFarland scale and seeded on a Mueller Hinton Agar plate supplemented with 5% defibrinated horse’s blood and 20 mg/L β-NAD (PlastLabor^®^, Brazil). Then, the following antibiotics disks (Oxoid^®^, Brazil) were transferred to the surface of the seeded plate: benzylpenicillin (1 U), ciprofloxacin (5 μg), clindamycin (2 μg), erythromycin (15 μg), linezolid (10 μg), tetracycline (30 μg), rifampin (5 μg) and trimethoprim-sulfamethoxazole (1.25/23.75 μg). Results were obtained after incubation in a 5% CO_2_ atmosphere at 35 °C ± 1 °C for 40–44 h. Additionally, benzylpenicillin susceptibility was determined using an *E*-test (bioMerieux^®^, Brazil), which provided a minimum inhibitory concentration (MIC) value.

The quality control of the tests was also carried out as recommended by the BrCAST document (Brazilian Committee on Antimicrobial Susceptibility Testing (BrCAST), 2024) and included the use of *Streptococcus pneumoniae* ATCC 49619.

### 2.3 Genome sequencing, assembling, and annotation

Bacterial genomic DNA was extracted with the MagNA Pure 24 Total NA Isolation (Roche Life Sciences^®^, Germany) according to the manufacturer’s instructions. WGS was performed by the Illumina^®^ MiSeq platform (California, USA). The preparation of libraries for sequencing used the commercial DNA Prep kit (Illumina^®^) following the manufacturer’s instructions, and the sequencing run was performed with MiSeq Reagent Kit v2 reagents (Illumina^®^), with 300 cycles, 2 × 151 – base pair paired-end, according to the manufacturer’s recommendations. Run quality control was done by including PhiX (Illumina^®^) in each run, and the median coverage was 50×. The quality of raw reads was initially assessed using FastQC v. 0.12.0 (Babraham Bioinformatics – *A Quality Control tool for High Throughput Sequence Data*) to identify potential issues such as adapter contamination, low base quality, or overrepresented sequences. Subsequently, Fastp v. 0.23.4^1^ was used to perform trimming and filtering of raw reads. Fastp was configured to: automatically detect and remove adapter sequences; trim low-quality bases (*Q* < 20) from both ends; discard reads shorter than 50 bp after trimming; and remove reads with more than 5% of ambiguous bases (N). In addition, poly-G and poly-X tails–common sequencing artifacts–were trimmed from the ends of reads, and overrepresentation analysis. Quality-filtered reads were used for downstream analysis, including genome assembly and annotation. The genome was assembled *de novo* using Unicycler v. 0.5.0. CheckM2 v. 1.0.1 (Chklovski et al., 2023) was used to evaluate the completeness and contamination. GUNC v. 1.0.5 was applied to check for chimerism, by taxonomic classification of the genes from each contig and estimating the adjusted clade separation score (CSS). An adjusted CSS value above 0.45 suggests contamination (Orakov et al., 2021). PlasmidFinder v. 2.1.6 (Carattoli et al., 2014) was used to check the presence of plasmids, Barnapp v. 0.9 (Quast et al., 2012) for completeness of rRNA genes, and QUAST v. 5.0.2 (Gurevich et al., 2013) for assembly statistics. Finally, PHASTER (Arndt et al., 2016) was applied to investigate the presence of prophage sequences. The genomes were annotated using the NCBI Prokaryotic Genome Annotation Pipeline (Tatusova et al., 2016) and deposited in GenBank under the accession number JAUOOX000000000.

### 2.4 Genomic taxonomy and phylogenetic analysis

The taxonomic classification of the bacterial isolate was performed using the Ribosomal Multilocus Sequence Typing (rMLST) database for species identification (Jolley et al., 2012), GTDB-Tk v. 2.3.0 (Chaumeil et al., 2022) and Type Strain Genome Server (TYGS).^2^ The closest reference genomes identified by TYGS were used to build an Average Nucleotide Identity (ANI) heatmap using PyANI v. 0.2.12 (Pritchard et al., 2016) with BLASTn v 2.5.0 (Tamura et al., 2021). DNA-DNA hybridization (DDH) was determined *in silico* using the Basic Local Alignment Search Tool (BLAST) method’s Genome-to-Genome Distance Calculator (GGDC) v.3.0. The results were based on recommended formula 2 (identities/HSP length) (Emms and Kelly, 2019). The genome was compared with the type strains of the most related species: *C. diphtheriae* NCTC 11397*^T^*, *Corynebacterium belfantii* FRC0043*^T^*, *Corynebacterium rouxii* FRC0190*^T^* *Corynebacterium pseudotuberculosis* ATCC 19410*^T^*, *Corynebacterium silvaticum* KL0182*^T^* and *Corynebacterium ulcerans* NCTC 7910*^T^*.

For phylogenetic inference, we used the closest type strains identified by TYGS or others that we consider important and that relate to our study and *Lawsonella clevelandensis* X1036 (GenBank accession number GCA_001293125.1) as an outgroup. Genomes were selected based on high sequencing coverage, prioritizing RefSeq assemblies and excluding those with significant contamination. The proteomes were downloaded using NCBI’s Datasets v. 15.2.0.^3^ The phylogenetic tree was built using OrthoFinder pipeline v. 2.5.5 (Emms and Kelly, 2019) with MAFFT algorithm (Katoh et al., 2002) for protein sequence alignment and FastTree (Price et al., 2010) for tree inference (parameter “-M msa”). The tree was visualized using iTOL v. 6 (Letunic and Bork, 2021).

### 2.5 MLST characterization, virulence factors, drug resistance genes, and CRISPR-Cas system identification

The sequence type (ST) was determined by *in silico* extraction of *atpA, dnaE, dnaK, fusA, leuA, odhA*, and *rpoB* sequences from WGS data using the Institut Pasteur MLST database.^4^ The sequences of these seven housekeeping genes were deposited in the Institut Pasteur MLST database. MLST alleles of *C. diphtheriae* strains available in the MLST database and isolated in Brazil and other countries were used to build a phylogenetic tree using IQ-TREE 2 (Minh et al., 2020).

The VFDB (Virulence Factor Database) determined the prediction of bacterial virulence factors and analyzed them using VFanalyzer (Liu et al., 2022). Additionally, PanViTa v. 1.1.3 (Rodrigues et al., 2023) was used to predict antimicrobial resistance genes (ARGs) and virulence genes using CARD (Comprehensive Antibiotic Resistance Database) and VFDB, respectively. In addition, the search for drug resistance and virulence genes was also performed using the gene functional annotation from the BlastKOALA server v. 3.1 (Kanehisa et al., 2016).

CRISPRCasFinder v. 1.1.2 was used to identify the genome’s CRISPR-Cas system. Only CRISPR arrays with evidence equal to 3 or 4 were included in the analyses (Couvin et al., 2018). The type of CRISPR-Cas cassette was determined following the nomenclature and classification previously described (Makarova and Koonin, 2015). Spacers from CRISPR arrays were extracted from CRISPRCasFinder outputs. Spacer sequences were analyzed for their identity in the CRISPR-Cas++ and CRISPRTarget v. 2 (Biswas et al., 2013) databases. In the CRISPR-Cas++ database, we used an *E*-value = 0.01. Spacer hits were selected from the CRISPRTarget v. 2 and CRISPR-Cas++ databases with a cut-off Identity Cover, according to Sangal et al. (2013). In CRISPRTarget v. 2, the cut-off score was the default parameter value. This database contains A CLAssification of Mobile Genetic Elements (ACLAME), Genbank-Phage, RefSeq-Plasmid, RefSeq-Viral, IslandViewer, PHAST, and Community Cyberinfrastructure for Advanced Microbial Ecology Research & Analysis (CAMERA) sequences.

### 2.6 Structural analysis of mutations

Initially, we compared the sequence obtained from the RNA polymerase Beta subunit (*rpo*B) of *C. diphtheriae* 46855 with a reference sequence no mutation (*C. diphtheriae* HC03; GenBank AEX78103.1) using BLAST. To analyze this mutation in the *rpoB* gene, putatively involved in resistance to rifampin, the *Mycobacterium tuberculosis rpoB* complex (5UHC_B) with rifampin (PDB:5UHC), and *C. diphtheriae rpoB* containing a serine at position 445 and asparagine at position 441 (*C. diphtheriae* HC03) were obtained by searching for similarity from target sequence 46855_*rpoB* (containing a phenylalanine at position 445 and tyrosine 441 obtained in this study) in the PDB and NCBI databases, respectively, using BLAST.

The *rpoB* of *C. diphtheriae* (named here as rpoB*-*N441Y-S445F for *C. diphtheriae* genome 46855 and rpoB-N441-S445 for reference no mutation *C. diphtheriae* HC03) were modeled with AlphaFold2 v. 2.3.0 (Jumper et al., 2021), refined using a web tool ModRefiner version 2018.03.22 (Xu and Zhang, 2011; Zhang and Skolnick, 2005) and evaluated with MolProbity version 4.5.2 (Williams et al., 1994). The molecular docking experiments were performed using Autodock Vina software v. 1.1.2 (Trott and Olson, 2010), following its docking protocol, that is, the addition of polar hydrogen and atomic partial charge signature with the Gasteiger method both proteins and ligand (rifampin). The 3D interaction maps were generated using Discovery Studio Visualizer software version 4.5 (Minh et al., 2020) and figures using Pymol v. 2.5.0 (The PyMOL Molecular Graphics System, Version 1.2r3pre, Schrödinger, LLC.), as well as for docking analysis.

The molecular dynamics simulation of the rpoB-N441Y-S445F-rifampin complex was conducted for 100 nanoseconds using the GROMACS 2023 package (Abraham et al., 2015) under standard *in vitro* conditions, including physiological temperature, pH, and pressure, with the CHARMM36 force field version July 2022 (Huang and MacKerell, 2013). The protein and ligand structures were prepared by protonation through CHARMM-GUI v. 3.8 web tools (Jo et al., 2008), and the ligand parameters were generated using the CGenFF web tool (Vanommeslaeghe and MacKerell, 2012). Root Mean Square Deviation (RMSD) plots were generated using Xmgrace software (Makarova and Koonin, 2015).

The stability analysis of rpoB-N441-S445 (reference no mutation) and rpoB*-*N441Y-S445F (present in *C. diphtheriae* 46855) was performed through the DynaMut2 web server^5^ (Rodrigues et al., 2021) that uses normal mode analysis methods (NMA) with graphs to capture the possible movement of the protein in the environment, highlighting the effects triggered by the mutation in the structure, in relation to stability and dynamics. For internal contacts evaluation the VTR web tool was used (Pimentel et al., 2021).^6^ This web tool searches for differences and similarities in pairs of residuals in contact comparing with analogous positions, based on cutoff and scoring. It also performs structural alignments between protein pairs through the standard parameters of the TM-align algorithm (Zhang and Skolnick, 2005). Thus, it was possible to measure the differences between both *C. diphtheriae rpo*B based on the mean AVD (Mean Vector Distance) of the corresponding contacts (Pimentel et al., 2021).

### 2.7 Biofilm formation capacity, virulence *in vivo* analysis and bacterial interaction

The biofilm formation capacity was performed according to Rocha et al. (2019) with modifications. Overnight cultures of *C. diphtheriae* 46855 in Brain Heart Infusion (BHI, Acumedia-Neogen, Brazil) broth were adjusted to a 0.5 McFarland standard (∼1 × 10^8^ cells/mL), and 200 μL were transferred to 96-well polystyrene microtiter plates. The plates were incubated at 37 °C for 1, 3, 6, and 24 h. After incubation, planktonic cells were discarded, and the wells were washed three times with sterile saline solution (0.9% NaCl) to remove unattached cells. Biofilms were fixed with methanol for 15 min and stained with 1% crystal violet for 20 min. Excess crystal violet was removed, and the wells were washed three times with saline. After air-drying, 200 μL of a 33% acetic acid solution was added to each well, and absorbance was measured at 600 nm. All experiments were performed in triplicate and repeated three times. The biofilm-forming capacity of the isolate was classified based on the criteria of Mathur et al. (2006) with modifications. Statistical differences were determined using ANOVA with Tukey’s *post-hoc* test.

*In vivo* virulence assay was performed according (Weerasekera et al., 2018, 2019) with modifications. For that, *C. diphtheriae* 46855 overnight cultures in BHI broth were adjusted to 1 × 10^10^ cells/mL and serially diluted to 1 × 10^3^ cells/mL (1:10 *v/v*). Groups of ten *Galleria mellonella* larvae, weighing 250–300 mg and free of melanization signs, were selected. Bacterial cells (10 μL) were injected directly into the hemocoel of each larva through the last right proleg using a Hamilton syringe. The larvae were incubated at 37 °C in the dark, and survival was monitored every 12 h over 10 days. The experiment was repeated three times, and statistical differences were determined using the log-rank test.

Bacterial interaction assays were performed using the A549 pneumocyte cell line (CCL-185™) based on protocols previously described (Cappelli et al., 2022) with modifications. The cells were maintained in Dulbecco’s Modified Eagle’s Medium – DMEM (Sigma Chemical Co^®^, USA) supplemented with 10% fetal calf serum (FCS) (Gibco BRL^®^, USA), 50 μg/mL gentamicin (Sigma Chemical Co^®^), and 0.5% L-glutamine (Sigma Chemical Co^®^) at 37 °C, in a 5% CO_2_ atmosphere.

For the quantitative analysis, A549 monolayers were cultivated on 96-well microplates (Corning, USA) from an initial count of 2.5 × 10^5^ cells/mL. Bacteria were grown overnight in Tryptic Soy Broth (TSB; Kasvi^®^, Spain) at 37 °C, suspended in DMEM (without supplementation) and used to infect the A549 monolayer for different periods (1, 2, 3, 4, and 6 h) with a multiplicity of infection (MOI) of 10. To determine the number of total and associated bacteria, at each infection period, supernatants were recovered, and the monolayers were washed 3 times with phosphate buffered saline (PBS; 0.01 M; pH 7.2) and lysed for 3 min with 0.1% Triton X-100 (Sigma-Aldrich Co^®^) in PBS. Aliquots from the supernatants and lysates were then diluted in PBS and plated on the surface of Columbia Agar Base (CAB; Kasvi^®^). To evaluate the number of viable intracellular bacteria, the monolayers were treated with 250 μg/mL amikacin (Sigma-Aldrich Co^®^) for 1 h and washed 5 times with PBS before lysing. Aliquots from these lysates were also diluted in PBS and plated on the surface of CAB plates. The assays were performed in 4 replicates and repeated three times.

Additionally, bacterial adherence pattern assays were performed by using semi-confluent A549 monolayers grown on glass coverslips with 13 mm diameter from the initial count of 1 × 10^5^ cells/mL. Three and 6 h post-bacterial infection (MOI 100), coverslips were washed with PBS, stained with a panoptical fast staining kit (Newprov^®^, Brasil) and examined by bright field microscopy with the Nikon eclipse 80i equipment (Nikon^®^, Japan). Adherence patterns were classified as follows: localized (LA), characterized by small clusters of bacteria resembling micro-colonies; diffuse (DA), bacterial cells randomly distributed on eukaryotic cells surfaces; aggregative (AA), characterized by clumps of bacteria with a “stacked-brick” appearance.

## 3 Results

### 3.1 Phenotypic characterization and antimicrobial susceptibility profile of the isolate

After incubation, the growth of white, opaque colonies, showing slight hemolysis, was observed. Gram-stained optical microscopy of these colonies showed Gram-positive bacillary forms arranged in pallid shapes with angular formations between cells. The MALDI-TOF MS identified this bacterial isolate as *C. diphtheriae* with a 99% probability. The API^®^ Coryne System identified the isolate as *C. diphtheriae* biovar *mitis* (biocode 1010324, 96%). The isolate was resistant to tetracycline (16 mm; cut-off resistance < 24 mm) and rifampin (13 mm; cut-off resistance < 24 mm). However, the isolate was found susceptible to clindamycin (30 mm; cut-off ≥ 15 mm), erythromycin (43 mm; cut-off ≥ 24 mm; *D*-test negative) linezolid (35 mm; cut-off ≥ 25 mm), and trimethoprim-sulfamethoxazole (32 mm; cut-off ≥ 23 mm). Susceptible with increased exposure to benzylpenicillin (22 mm; cut-off 12–49 mm) and ciprofloxacin (36 mm; cut-off 24–49 mm). Susceptibility with increased exposure to benzylpenicillin was confirmed by a minimum inhibitory concentration (MIC) of 0.064 mg/mL (cut-off: 0.002–1 mg/mL). The isolate was named *C. diphtheriae* 46855. Another colony was isolated from the primary culture and identified by MALDI-TOF as *Pantoea agglomerans* (99% probability).

### 3.2 Genome features, taxonomy, and phylogeny

*C.* 46855 has a genome size of 2,448,501 bp, with a G + C content of 53.5%, 2338 CDSs, 48 tRNA, and 3 rRNA complete genes. Completeness and contamination were estimated as 100% and 0.56%, respectively. No plasmid and chimerism were identified. Isolate 46855 presented one incomplete prophage with 15 proteins and a G + C content of 55.10% (Supplementary Table 1). Detailed information about the genome is also shown in Table 1. The isolate was classified as *C. diphtheriae* due to its rMLST profile, DDH *in silico* of 83.6% (Supplementary Table 2), and ANI of 98% in comparison to *C. diphtheriae* type strain NCTC11397*^T^* (GTDB-Tk).

**TABLE 1:** General features of genome sequences of *Corynebacterium diphtheriae* 46855 isolate.

Strain	46855
Completeness (%)	100
Contamination (%)	0.56
Coverage	50×
Chimerism	No
Estimated genome size (bp)	2,2448,501
GC (%)	53.5
Contigs	54
N50	126,429
L50	6
Number of CDS	2,338
rRNAs	3
tRNAs	48
Prophages	1

Bp, base pair; GC, guanine-cytosine content; N50, sequence length of the shortest contig at 50% of the total assembly length; L50, count of smallest number of contigs whose length sum makes up half of genome size; CDS, coding sequence.

**FIGURE 1:**
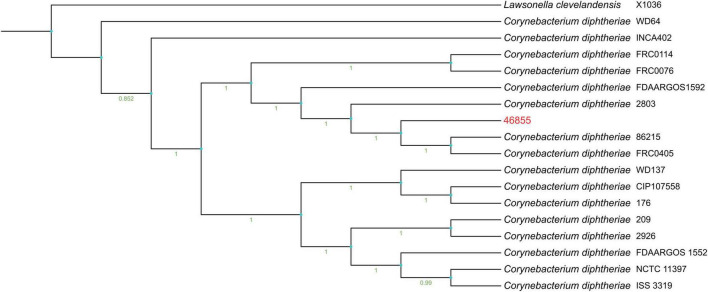
Phylogenetic tree genome shows the bootstrap percentage with 1000 bootstraps on the tree branches, ranging from 0.951 as the lowest percentage to 1 as the highest one. The outgroup was represented by *Lawsonella clevelandensis* X1036.

Supplementary Figure 1 shows a heatmap of ANI, including strain 46855 and the closest reference genomes identified by TYGS. Figure 1 presents the phylogenetic tree showing the position of *C. diphtheriae* 46855 among other *C. diphtheriae* strains.

### 3.3 MLST characterization, virulence factors, drug resistance genes, and CRISPR-Cas system analysis

Analyses of the 7 housekeeping genes showed that they belong to ST-925. The allelic profile is 2-1-37-19-24-103-4 for *atpA, dnaE, dnaK, fusA, leuA*, *odhA*, and *rpoB* genes, respectively. A phylogenetic tree based on the sequences of the *loci* used in the MLST scheme of the *C. diphtheriae* complex, including ST attributed to strains isolated in Brazil and other countries, is presented in Supplementary Figure 2. *C. diphtheriae* 46855 clustered with other strains isolated from different regions of Brazil (*C. diphtheriae* 799; ST-175; Brazil – RJ).

In the genome of *C. diphtheriae* 46855, 44 virulence-associated genes were detected, including three pilus gene clusters usually identified in diphtheria bacilli: *spaABC*, *spaDEF*, and *spaHIG*, encoding the pili SpaA, SpaD, and SpaH, respectively. Three genes coding surface-anchored pilus proteins (*sapA, sapD*, and *sapE*) and three non-fimbrial adhesins (DIP0733, DIP1621, and DIP1281) were also detected. Genes involved in iron uptake systems, such as ABC transporters (*fagABC* operon and *fagD* gene), ABC-type heme transporter (*hmuTUV* cluster, and hemin-binding proteins HtaA, HtaB and HtaC proteins), Ciu iron uptake and siderophore biosynthesis system (*ciu*A*BCDE* cluster), and Siderophore-dependent iron uptake system (*irp6ABC* operon) could also be found. Additionally, a gene involved in post-translational modification was identified (protein product DIP1880 - MdbA). Additionally, *sigA*, *embC, aftB, mptC*, and *dtxR* were detected. More details are presented in Supplementary Table 3. About ARGs, the genomic analysis revealed the presence of 4 genes: *sul1*, *tet*(33), *rbpA*, and *rpoB2*, and we did not find mutations in the *gyrA* gene. Finally, BLAST analysis did not reveal the presence of the *rbp* gene.

The CRISPRCasFinder server identified the Type I-E CRISPR-Cas system (*cas5*, *cas7*, *cse2*, *cas6*, *cas3*, *cas1*, *cas2*) and two CRISPR arrays (evidence levels 3 and 4). According to the databases, 9 spacers were found between the two CRISPR arrays, and only 1 unknown spacer was found. CRISPRTarget found hits for eight spacers. From the CRISPR array located in another contig (contig 3), five spacer sequences shared identities with different phages, such as *Corynebacterium* and *Rhocococcus* phages, with identity cover scores between 0.89 and 1.00. For the CRISPR array adjacent to the *cas* genes, the CRISPRTarget database found hits only for Brazilian *C. diphtheriae* strains, with identity cover scores of 1.00. CRISPRCas + + database found hits for seven spacers that shared identities only Cas-type I-E and II-C from Brazilian *C. diphtheriae* strains, with similarity values ranging from 96% to 100%. Further details can be found in Supplementary Table 4.

### 3.4 Structural analysis of mutations

5UHC_B *Mycobacterium tuberculosis rpoB* (Lin et al., 2017) shares 76% identity with rpoB*-*N441Y-S445F (*C. diphtheriae* 46855) and 75.89% with rpoB-N441-S445 (reference no mutation), while these two share 99.83 with each other. The analysis with MolProbity suggests that the models generated with AlphaFold2 are viable, presenting 97% of the residues in favorable positions, 99.8% in allowed regions and Molprobity 2.3 scores in both structures (Supplementary Figure 3).

DynaMut2 analysis suggest based on Predicted Stability Change (ΔΔ GStability) suffers an alteration of −0.36 kcal/mol, implying lower stability in rpoB*-*N441Y-S445F. In rpoB-N441-S445 Asparagine-441 acts on the stability of the protein by performing six clash interactions, while also performing three Van Der Waals (VDW) interactions, five hydrogen bonds and ten polar interactions. Similarly, when serine-445, this residue performs four clash interactions, one carbon bond, four VDW interactions, 10 hydrogen bonds and 11 polar interactions.

In relation to rpoB*-*N441Y-S445F, there is a difference in the number of interactions performed, with five clash interactions, two VDW interactions, three aromatic stacking interactions, five polar interactions, four hydrogen bonds and fifteen hydrophobic interactions only performed by Tyrosine at position 441. Phenylalanine, on the other hand, had eight clash interactions, five VDW interactions, twelve aromatic stacking, thirteen polar interactions, six hydrogen bonds and twenty-one hydrophobic interactions. The residues involved in each interaction according to DynaMut2 can be seen in Supplementary Table 5.

VTR analysis shows that Tyrosine at position 441 induces the formation of hydrophobic interactions with Phenylalanine at position 445 in rpoB*-*N441Y-S445F, also found two contact matches formed by interactions originally present in rpoB-N441-S445 partially conserved in rpoB*-*N441Y-S445F through interactions carried out by Y-441 with F-445 (Supplementary Figure 3).

VTR alignment of the structures obtained an RMSD score of 0.68, indicating that they are very similar. There was an increase in aromatic stacking with the number of hydrophobic interactions formed in rpoB*-*N441Y-S445F rose to 2245 whereas 2237 were formed in rpoB-N441-S445. Meanwhile, there was a drop of 09 hydrogen bonds, 43 attractive forces and 72 repulsive forces, indicating that rpoB*-*N441Y-S445F performs fewer interactions than rpoB-N441-S445, however, 68.73% of the contacts are common in both proteins.

**FIGURE 2:**
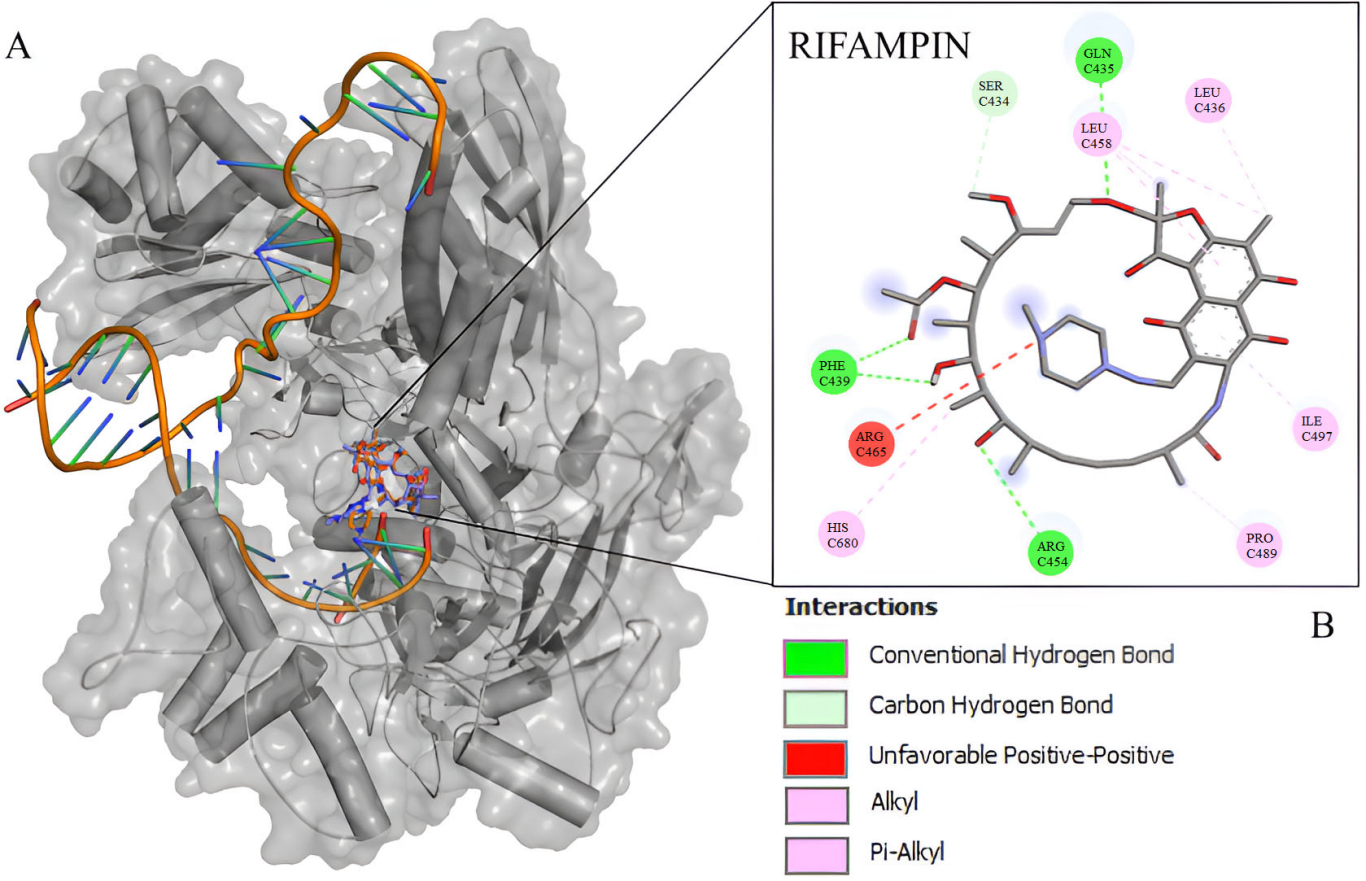
5UH_B redocking with rifampin. **(A)** *Mycobacterium tuberculosis* beta subunit with RNA and rifampin native (orange) and redocking (blue). **(B)** 2D interaction map between rifampin and 5UHC_B binding site residues.

**FIGURE 3:**
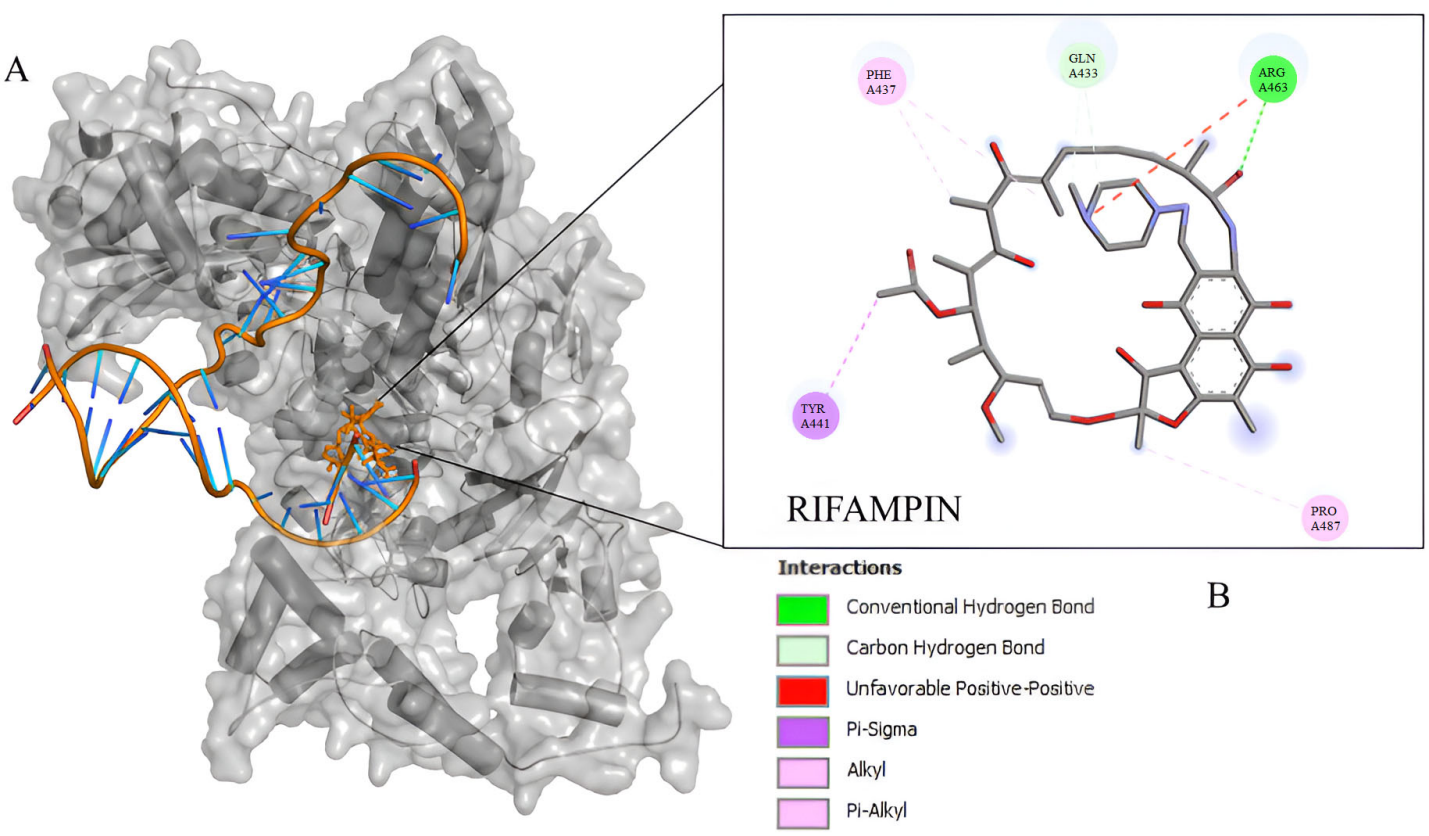
Docking experiment *Corynebacterium diphtheriae rpoB* and rifampin. **(A)** Beta subunit complexed with rifampin in native position (orange) and docked position (blue). **(B)** 2D interaction map between rifampin and rpoB-N441Y-S445F binding site residues.

The docking experiment suggests a higher affinity of rifampin with *M*. *tuberculosis rpoB* (Supplementary Table 6). Compared to rpoB*-*N441Y-S445F based on the energy of affinity estimation and the greater number of hydrogen bonds formed (Figure 2). The top one pose docking score for rpoB-N441-S445 with rifampin was −8.3 kcal/mol which is slightly larger than in rpoB*-*N441Y-S445F probably caused by the hydrogen bond formed with asparagine at position 441 (Figure 3) in rpoB-N441-S445 rifampin complex while was formed a Pi-sigma in rpoB*-*N441Y-S445F rifampin complex.

**FIGURE 4:**
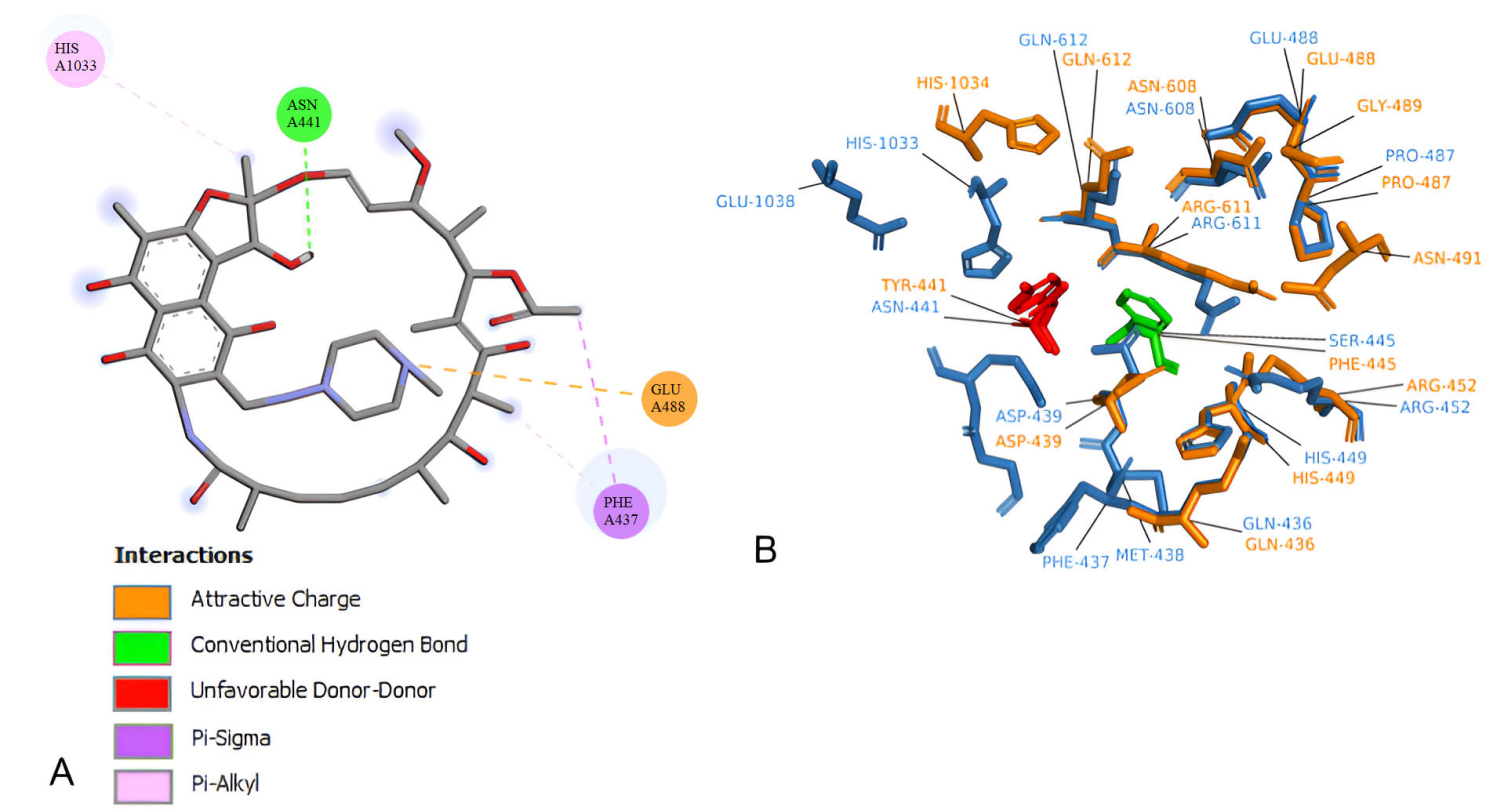
*Corynebacterium diphtheriae* rpoB-N441-S445 Binding site residues. **(A)** 2D interaction map between rifampin. **(B)** rpoB-N441-S445 (blue) and rpoB-N441Y-S445F (Orange) Superposition in rifampicin binding site at 5 angstroms contact, highlighting amino acid substitutions.

Also docking experiments indicate that rifampin established contact at a distance <5 angtros with the same types of amino acids, mostly in 5UHC_B and rpoB*-*N441Y-S445F complex (Figure 4). It was also observed that asparagine 443 in 5UHC_B complex occupies the same position as tyrosine 441 in rpoB*-*N441Y-S445F (Supplementary Figure 4). It is important to note that there are no interactions with Phenylalanine or Serine-445 (Serine-447 in 5UHC_B) in rpoB*-*N441Y-S445F and rpoB-N441-S445 with rifampin complex, respectively. These residues are in proximity with bidding sites residues but at the same time inaccessible interactions with rifampin, so phenylalanine substitution for serine does not interfere directly with rifampin affinity in this case.

The N441-to-Y441 substitution in rpoB-N441Y-S445F alters rifampin orientation and decreases the stability of the *rpoB*-rifampin complex, likely reducing rifampin’s effectiveness against *C. diphtheriae*. Furthermore, a 100-nanosecond molecular dynamics simulation shows that the rpoB-N441Y-S445F-rifampin complex loses stability, with rifampin vacating the binding site within 30 nanoseconds (Supplementary Figure 5).

### 3.5 Biofilm formation capacity, virulence *in vivo* analysis and bacterial interaction

**FIGURE 5:**
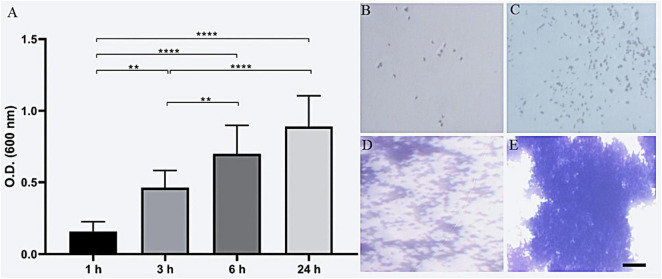
Biofilm biomass formation of *Corynebacterium diphtheriae* 46855 by crystal violet method **(A)**; representative biomass formation after 1 h **(B)**, 3 h **(C)**, 6 h **(D)** and 24 h **(E)**. ANOVA with Tukey’s post-test. **p < 0.01, ****p < 0.001. Scale bar = 200 μm.

Analysis of the biofilm-forming capacity of *C. diphtheriae* 46855 revealed its ability to adhere to abiotic surfaces (polystyrene) after 1 h of incubation. The biomass increased progressively after 3, 6, and 24 h of incubation (Figure 5). According to the classification by Mathur et al. (2006) for biofilm formation by *Staphylococcus* spp., the isolate was categorized as a strong biofilm former.

**FIGURE 6:**
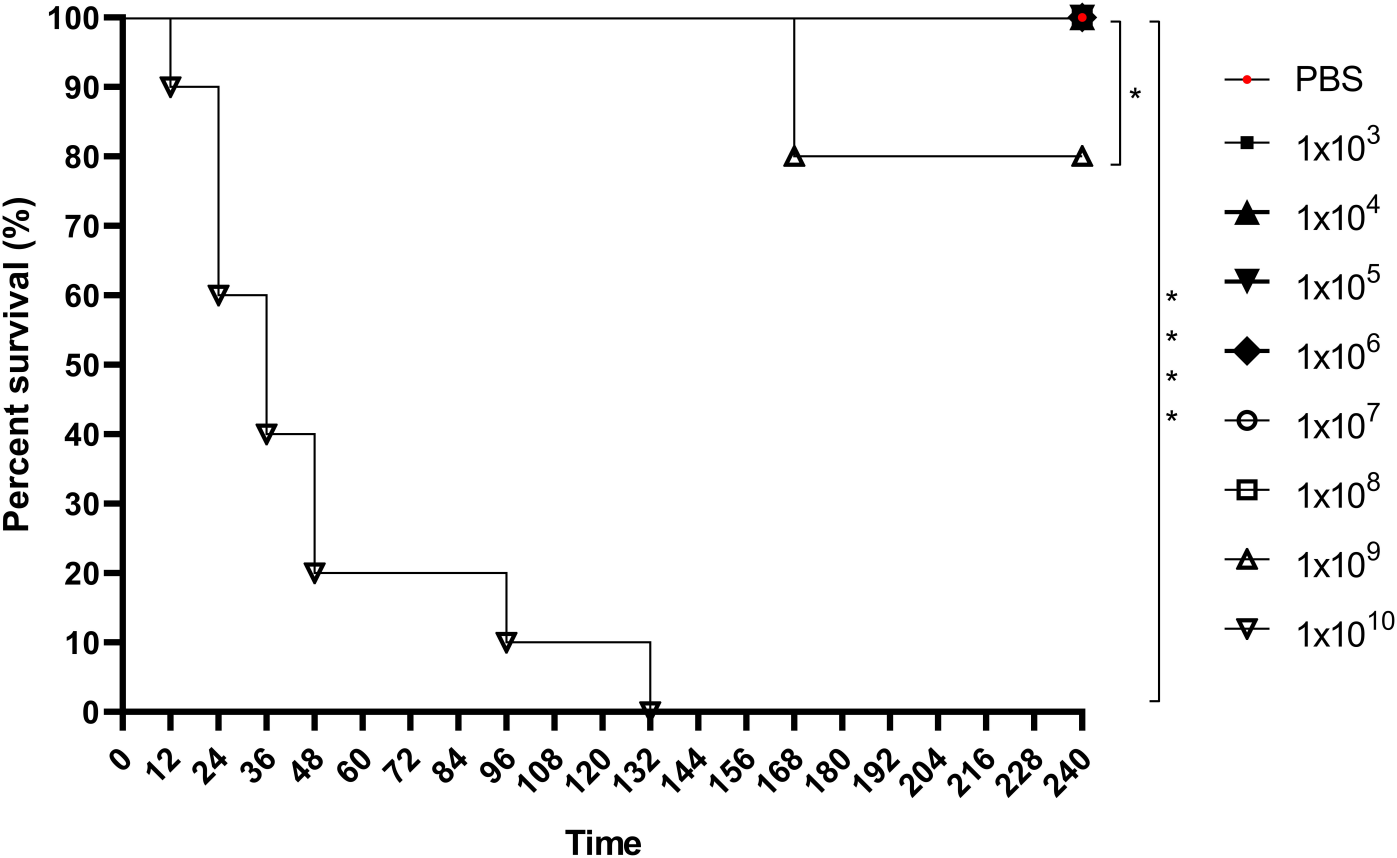
Keplan-Meier’s survival curve of *Galleria mellonella* larvae infected with 1 × 10_3_, 1 × 10_4_, 1 × 10_5_, 1 × 10_6_, 1 × 10_7_, 1 × 10_8_, 1 × 10_9_, and 1 × 10_10_ cells/mL of *Corynebacterium diphtheriae* 46855. Data showed that the 1×10_9_ and 1×10_10_ cells/mL inoculum resulted in significantly higher mortality than less concentrated inoculum and control condition (PBS). Logrank test *p < 0.05; ****p < 0.001.

The *G. mellonella* survival assay demonstrated that an inoculum of 1 × 10^9^ *C. diphtheriae* 46855 cells/mL caused 20% larval mortality after 168 h. A higher inoculum (1 × 10^10^ cells/mL) resulted in 100% mortality after 132 h, with 80% mortality occurring within the first 48 h (Figure 6), highlighting the virulence of this bacterial isolate in the invertebrate model.

**FIGURE 7:**
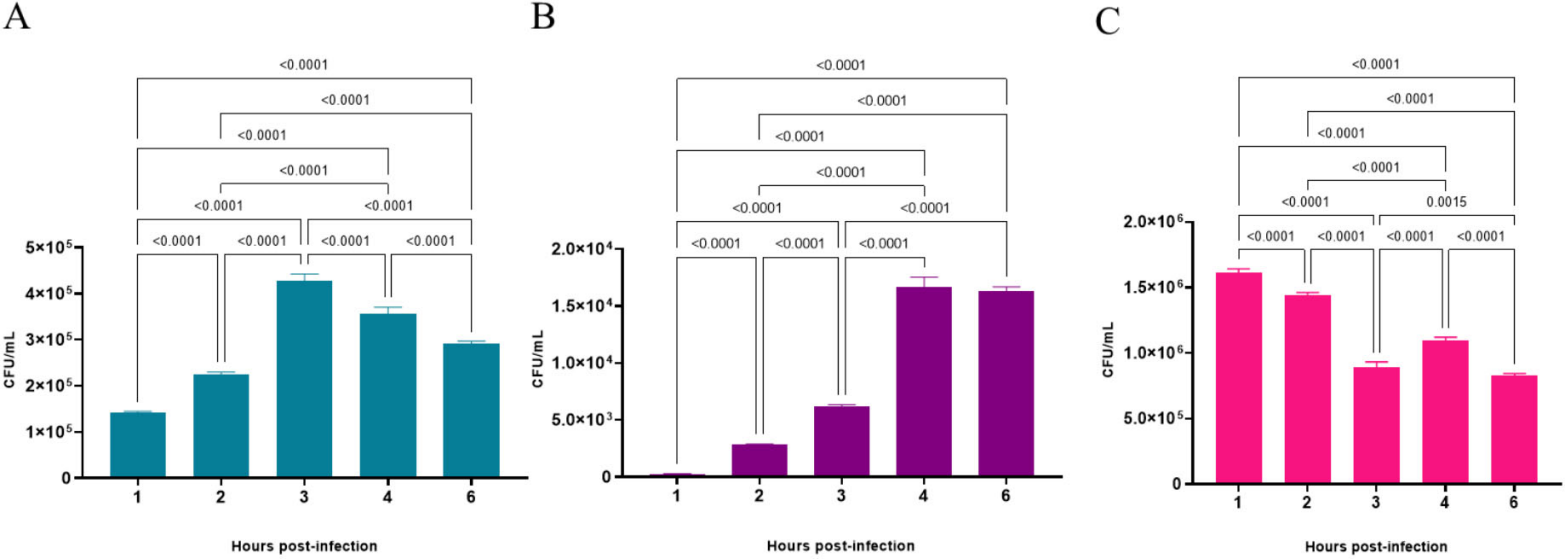
Number of viable bacteria of *Corynebacterium diphtheriae* 46855 strain associated with **(A)**, internalized by **(B)** and in the supernatant of **(C)** A549 monolayers in different periods. Data presented as the mean ± standard deviation of three independent experiments carried out in 4 replicates. Data were considered statistically different when *p* ≤ 0.05. CFU, colony forming units.

The results of the quantitative analysis of bacterial interaction assays with A549 cell line are presented in Figure 7. They revealed that *C. diphtheriae* 46855 can associate with pneumocyte monolayers since viable bacteria were found adhered to and inside A549 cells. As observed in Figure 7A, although viable bacteria adhered to A549 cells were found in all time evaluated, the highest number was detected 3 post-infection (3.6 ± 0.15 × 10^5^), which corresponds to an adhesion percentage equal to 32.5%. Regarding viable bacteria in the intracellular compartment of pneumocytes, the highest numbers of bacteria were found at 4 (1.7 ± 0.08 × 10^4^; invasion percentage = 4.7%) and 6 h of infection (1.6 ± 0.04 × 10^4^; invasion percentage = 5.6%) (*p* > 0.5134) (Figure 7B). During all the experiment, *C. diphtheriae* 46855 remained viable in the supernatant. However, the number of bacteria varied from time to time due to adhesion and internalization processes (Figure 7C).

**FIGURE 8:**
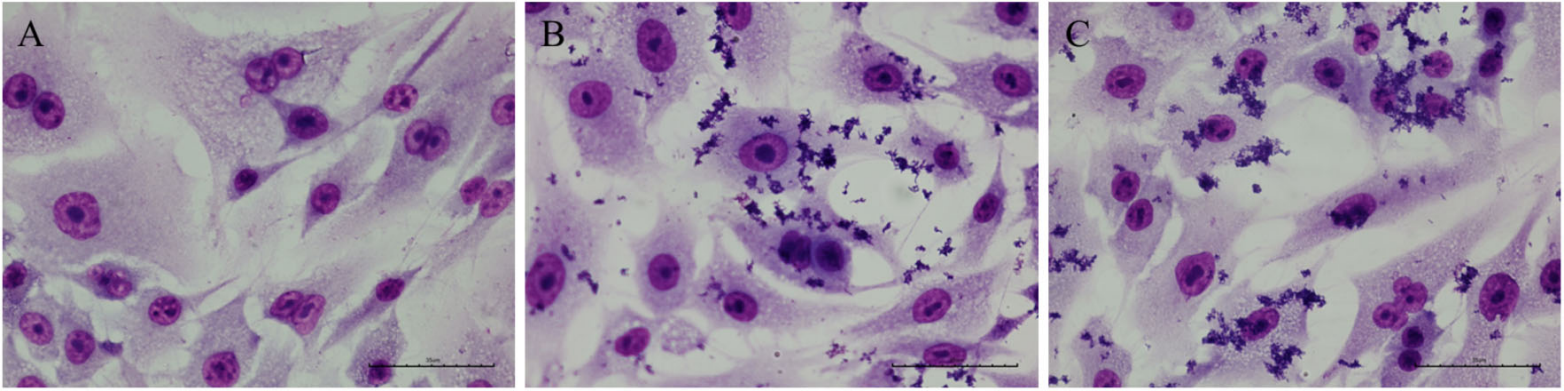
Micrographs of A549 cells infected with the *Corynebacterium diphtheriae* 46855 strain. **(A)** Non-infected control; **(B)** 3 h post-infection; **(C)** 6 h post-infection. Magnification ×400. Scale bar equal to 35 μm.

The microscopic analysis of A549 cells infected with *C. diphtheriae* 46855 revealed an AA adherence pattern, which is characterized by clumps of bacteria with a “stacked-brick” appearance, in both periods evaluated (Figure 8).

## 4 Discussion

We describe a skin infection caused by *C. diphtheriae* in a teenager residing in Northeast Brazil. The values obtained for ANI were consistent and above the proposed cut-off point for the species limit (95% ∼ 96%) (Dangel et al., 2020; Kim et al., 2014). The strain in this study showed DDH values above the limit (70%) for species definition (Chun et al., 2018). Contamination in microbial studies refers to unintended microbial DNA from sources like water, PCR reagents, and DNA extraction kits that infiltrate samples during preparation (Salter et al., 2014). We detected a low contamination rate of 0.56%, ensuring our DNA’s quality and our analyses’ accuracy.

Recent outbreaks of diphtheria in the Maranhão and Pernambuco states in 2010 and 2015, respectively, highlight the vulnerability of the Brazilian Northeast region (Sangal et al., 2013; Santos et al., 2015). Despite complete vaccination, the individual was affected, confirming that cases of non-toxigenic *C. diphtheriae* strains may occur and represent significant health threat, even in vaccinated patients with ulcerated lesions (Araújo et al., 2023; Batista Araújo et al., 2021; Sangal and Hoskisson, 2020). The difficulty in clinically distinguishing *C. diphtheriae* infections from other skin infections further emphasizes the importance of ongoing surveillance (Mattos-Guaraldi et al., 2003).

Our isolate is genetically related to *C. diphtheriae* strains 86215 and FRC0405, belonging to ST-536, isolated in Rondônia, Brazil (Araújo et al., 2023), and French Guiana (Hennart et al., 2020), respectively (Figure 1 and Supplementary Figure 2). It shares a common ancestor with strain FRC0076, attributed to ST-212, and has been observed in strains from Germany, France, the USA, and Pernambuco (Brazil), according to the MLST database. The genomic analysis identified our cutaneous isolate as ST-925, a novel sequence type not previously reported in the literature, closely related phylogenetically to ST-175, isolated in Rio de Janeiro from a cutaneous infection. ST-925 differs from ST-175 only in the *odhA* allele (103 vs. 3, respectively), as per the MLST database. ST-925 also shares a common ancestor with Brazilian strains isolated in Minas Gerais (ST-647) from a case of cutaneous infection by a penicillin-resistant *C. diphtheriae* strain in a patient with diabetes mellitus (Batista Araújo et al., 2021), as well as strains involved in recent diphtheria outbreaks in Maranhão (ST-176) (Santos et al., 2015) and Pernambuco (ST-643). Diphtheria remains endemic in subtropical and tropical countries like Brazil, influenced by human host factors, socioeconomic conditions, and public health policies such as universal vaccination (Batista Araújo et al., 2021; Mattos-Guaraldi et al., 2003). The individual in this study exhibits precarious socioeconomic conditions. There is no confirmation of travel history or migration, which are critical factors in considering the spread of the causative agent.

In 2022, cases of diphtheria were recorded in Germany and several European countries, including Austria, Belgium, France, Norway, Switzerland and the United Kingdom (Badenschier et al., 2022). In Australia, cases increased by around 300% in North Queensland (Graham et al., 2023), and in Pakistan there were 45 deaths among children and adolescents (Aaqil et al., 2023). In 2023, the Nigerian Ministry of Health declared an outbreak with 25 deaths, mainly among children (Shariff et al., 2018). These events indicate a global trend of diphtheria re-emergence, attributed to falling vaccination rates during the COVID-19 pandemic (Hirabayashi, 2020; Saxena et al., 2020) and increased migration from countries with low vaccination coverage. Prevention requires monitoring of toxigenic and non-toxigenic *C. diphtheriae*, as importation of toxigenic strains or lysogenization of local strains by corynebacteriophages can initiate outbreaks (Bernard and Funke, 2015). Non-toxigenic strains, associated with serious diseases not preventable by vaccines, have been isolated and are considered emerging pathogens. Complete genomic sequencing, as performed on our isolate, is essential to detect virulent clones and antimicrobial resistance, contributing to the prevention of outbreaks (Chorlton et al., 2020; Setiawaty et al., 2022).

Analysis of WGS data with VFanalyzer and PanViTa identified resistance genes, including *sul1*, related to sulfonamide resistance (Chaturvedi et al., 2021), and *tet*(33), associated with tetracycline resistance (Markley and Wencewicz, 2018), corroborating the results of in-house susceptibility testing *in vitro*. Sulfamethoxazole was tested in combination with trimethoprim, explaining the observed susceptibility. No mutations were found in the *gyrA* gene, responsible for resistance to quinolones in corynebacteria (Ramos et al., 2020), confirming the susceptibility to ciprofloxacin. We observed resistance to rifampin *in vitro*, with the presence of the *rpoB2* and *rbpA* genes; previous studies have linked rifampicin resistance in *C. diphtheriae* to mutations in the *rpoB* gene. Furthermore, *rpoB2* appears to have a similar function to that observed in *Nocardia* spp., suggesting resistance (Hennart et al., 2020; Ishikawa et al., 2006; Luo et al., 2023; Meng et al., 2023; Viana et al., 2023). Given the need for more conclusive studies on rifampicin resistance in *Corynebacterium* spp., we carried out elaborate structural analyses.

Regarding the structure of the protein arranged in three-dimensional space, it is clear from all the results described here that both structures (rpoB-N441-S445 – reference no mutation and rpoB*-*N441Y-S445F – *C. diphtheriae* 46855) are very similar, although the presence of the two substitutions induces the formation of different interactions and changes the conformation of some residues. This data is in line with what is seen in relation to protein structure and function, since proteins that perform functions tend to be conserved between species, even expanding into different phyla (Chlenov et al., 2005). Thus, we point out that the Beta subunit of RNA polymerase does not tend to affect the primary function of this protein complex.

In *Mycobacterium leprae* the exchange of an arginine for a tyrosine at position 441 induces changes in the binding pocket by introducing an aromatic side chain into the pocket, which destabilizes the hydrogen-binding interactions with rifampin (Vedithi et al., 2018). Point mutations in this region, known as rifampin-resistance-determining region (RRDR), usually comprising the positions 410–480 in the codon are associated with resistance to carrying structural and physico-chemical changes at the rifampin binding site (Amusengeri et al., 2022). This substitution also led to a loss of hydrogen bonds and consequently turned the rifampin binding site reduction in hydrophilicity. Similarly, the substitution of asparagine 441 in *rpoB* by a tyrosine in this study ends up contributing to the development of a reduction in hydrophilicity environment due to the hydrophobic nature of the aromatic ring present in tyrosine. Furthermore, the introduction of the aromatic ring of Tyrosine may introduce the steric hindrance. The same reasoning can be applied to serine-445 substitution for phenylalanine, this last, also, contains an aromatic ring, which implies a change in amino acid interactions in binding site region and, consequently, some degree of change in conformation affecting affinity for rifampin. Thus, these changes can impact the orientation of rifampin at the binding site and lead to resistance as highlighted by Vedithi et al. (2018). Thus, the substitution of asparagine 441 with tyrosine affected the hydrogen bonds between rifampin and *rpoB* of *C. diphtheriae* 46855, influencing the orientation of rifampin in the pocket, as observed through the docking experiment. New insights into antimicrobial resistance are even more relevant given the scenario that the indiscriminate use of antibiotics in empirical therapies contributes to the increase in resistant strains, and *C. diphtheriae* exhibits varying resistance profiles that exacerbate this global concern (Husada et al., 2019; Markley and Wencewicz, 2018).

Our isolate possesses several virulence genes, including those encoding SpaA fimbriae (*spaABC* and *srtA* cluster), essential for adhesion to pharyngeal epithelial cells (Broadway et al., 2020). Furthermore, genes encoding proteins such as DIP0733 (cell adhesion and invasion), DIP1281 (surface organization) and DIP1621 (invasive hydrolase) are important for the establishment of infections (Antunes et al., 2015; Kolodkina et al., 2011; Ott et al., 2020; Sabbadini et al., 2012; Weerasekera et al., 2018). Lipoglycans, such as CdiLAM, may facilitate initial adhesion, although their role in cutaneous infections is minor (Mishra et al., 2011; Moreira et al., 2008). Genes *aftB*, *embC* and *mptC* contribute to cell wall structure and adhesion, reinforcing the invasive potential and resistance of *C. diphtheriae* (Jankute et al., 2018; Yimcharoen et al., 2022). The *sigA* gene, also identified in our isolate, encodes the sigma A factor, which is known to be essential for the initiation of transcription under stress conditions and different growth phases in *Corynebacterium* spp. (Escorcia-Rodríguez et al., 2021). These factors support the relevance of monitoring virulence and resistance in circulating strains, improving understanding of infection dynamics and therapeutic response.

Iron acquisition is essential for *C. diphtheriae* virulence, which must overcome host iron sequestration (Billington et al., 2002; Lyman and Peng Schmitt, 2018). Our isolate presented the *fagABC* operon and the *fagD* gene for iron uptake, the *hmuTUV* cluster, and the genes encoding heme-binding proteins (HtaA, HtaB, HtaC) involved in heme iron acquisition (Allen and Schmitt, 2009, 2011, 2015; Drazek et al., 2000). The *ciuABCD* cluster and the *ciuE* gene for transport and synthesis of siderophores, essential in low iron conditions, and the *irp6*ABC operon, whose inactivation impairs iron uptake by corynebactin, were also identified (Kunkle and Schmitt, 2005; Qian et al., 2002). These systems are fundamental for adaptation in the host, suggesting therapeutic targets. The *dtxR* gene, which product regulates iron metabolism and represses genes such as *irp6* and *ciu* via Fe^2+^ (Kunkle and Schmitt, 2005; Qian et al., 2002) was also detected. The oxidoreductase *mdbA*, essential for fimbriae assembly and toxin production (Reardon-Robinson et al., 2015), reinforcing virulence of *C. diphtheriae* 46855 even in the absence of the *tox* gene, as well as indicating adaptive and survival capacity in iron-poor environments, an essential factor for virulence and persistence in the host. The findings also suggest an invasive potential, highlighting the importance of monitoring.

CRISPR-Cas systems provide adaptive immunity in bacteria and archaea against exogenous DNA (Makarova et al., 2020). The CRISPR-Cas type I E in *C. diphtheriae* is a sophisticated adaptive defense system, providing specific protection, immune memory, and helping in the genetic characterization of strains (Sangal et al., 2013). The Type I-E CRISPR-Cas system is well-documented in *Corynebacterium* species (Cheleuitte-Nieves et al., 2018; Guimarães et al., 2016; Parise et al., 2018; Ramos et al., 2023), with variants of Type I-E and Type II-C also present in *C. diphtheriae* (Sangal et al., 2013). The canonical Type I-E system includes the gene sequence *cas3*, *cse1*, *cse2*, *cas7*, *cas5*, *cas6*, *cas1*, and *ca*s*2* (Allen and Schmitt, 2009). In *C. diphtheriae*, identified variants include Type I-E-a (*cas3*, CRISPR array, *cse1*, *cse2*, *cas7*, *cas5*, *cas6*, *cas1*, *cas2*) and Type I-E-b (*cas5*, *cas7*, *cse2*, *cse1*, *cas6*, *cas3*, *cas1*, *cas2*) (Sangal et al., 2013). Several variants coexist with spacer arrays inserted between genes such as c*as3* and *cse1*, suggesting horizontal transfer (Sangal et al., 2013). In strain 46855, we observed a Type I-E variation like Type I-E-b but lacking the *cas* gene *cse1* from the effector complex (*cas5*, *cas7*, *cse2*, *cas6*, *cas3*, *cas1*, *cas2*). Recognition of DNA targets requires crRNA complementarity and recognition of the protospacer-adjacent motif (PAM) by *cse1* (Gleditzsch et al., 2019), suggesting that the Type I-E variant in strain 46855 may be less efficient or non-functional. The diversity of spacer sequences reflects historical invasions by various phages and *Corynebacterium* species, highlighting the evolutionary and functional importance of this bacterial immunity system.

The ability of bacteria to adhere to surfaces and grow as biofilms is closely associated with persistent infections and treatment challenges, rendering biofilm-forming bacteria a significant clinical concern (Park et al., 2024; Qiu et al., 2020). Biofilm formation by *C. diphtheriae* has been poorly characterized; however, Gomes et al. (2009) reported a strain isolated from a nephrostomy catheter-related infection, suggesting its potential for adhesion to medical devices. Additionally, Sun et al. (2022) demonstrated heterogeneity in biofilm formation capacity among *Corynebacterium* species. In this study, we observed a progressive increase in biofilm formation by *C. diphtheriae* 46855 over time, culminating in strong biofilm formation after 24 h of incubation at 37 °C. The biofilm formation ability enhances antimicrobial resistance and enables the bacteria to persist under environmental stress (García-Betancur et al., 2017; Rogers et al., 2010). Understanding *C. diphtheriae* biofilm formation and its associated virulence factors could inform strategies for pathogen control.

The *G. mellonella* model is widely used to study bacterial pathogenicity (Cools et al., 2019; Ménard et al., 2021). In this study, we demonstrated that an inoculum with *C. diphtheriae* (1 × 10^10^ cells/mL) kill all the larvae tested. Weerasekera et al. (2018, 2019) identified the role of *rbp* genes in *C. diphtheriae* virulence using this model, with a 3 × 10^9^ cells/mL inoculum reducing the health index of larvae, demonstrating that model as suitable to *C. diphtheriae* studies. We did not find the *rbp* gene in our isolate, however our findings demonstrate the virulence of *C. diphtheriae* 46855 *in vivo* and highlight the importance of virulence factors identified in genome through bioinformatics analyses.

Although the nasopharynx is the preferred site of *C. diphtheriae* infections, cases of pneumonia, especially by non-toxigenic strains, have been reported (Mattos-Guaraldi et al., 2001; Zhou et al., 2022). The development of pneumonia and other invasive infections may occur due bacteremia, which has been associated to some risk factors, such as the colonization/infection by the microorganism (Romney et al., 2006; Zasada, 2013). Considering that *C. diphtheriae* 46855 is non-toxigenic, was isolated from a cutaneous infection case, and carries several genes for adhesins, sortases and invasins, we decided to investigate its abilities to adhere to and invade pneumocytes. *C. diphtheriae* 46855 showed remarkable adhesion and invasion abilities. Additional studies should be performed to identify and characterize the factors accountable for these mechanisms.

This study has certain limitations that should be considered. Most notably, the analyses were conducted on a single non-toxigenic *C. diphtheriae* strain, which limits the extent to which our findings can be generalized to other isolates or epidemiological settings. Although we performed comparative genomic analyses with phylogenetically related strains, the conclusions regarding virulence potential and antimicrobial resistance mechanisms, may not fully represent the broader population of *C. diphtheriae*. Additional studies involving a larger and more diverse set of non-toxigenic isolates are necessary to confirm and expand upon these observations.

Data obtained in the present work highlight that non-toxigenic *C. diphtheriae* strains isolated from skin should not be neglected since they may carry antimicrobial resistance genes in addition to important virulence factors that could promote the establishment of bacteria in other human sites and favor the development of severe infections, such as pneumonia. Moreover, it is important to note that non-toxigenic *C. diphtheriae* strains can be lysogenized and become able to produce the DT. Thus, these strains should also be monitored and investigated independently from the site of isolation.

## Data Availability

The datasets presented in this study can be found in online repositories. The names of the repository/repositories and accession number(s) can be found in the article/Supplementary material.

## Footnotes

1
https://github.com/OpenGene/fastp

2
https://tygs.dsmz.de/

3
https://github.com/ncbi/datasets

4
https://bigsdb.pasteur.fr/diphtheria/

5
https://biosig.lab.uq.edu.au/dynamut2/

6
http://bioinfo.dcc.ufmg.br/vtr/index.php
