# Supplementary material for: Virulence and mutations analysis based on the whole genome of a Brazilian Corynebacterium diphtheriae strain isolated from a cutaneous infection
Source: Front Microbiol. 2025 Oct 15;16:1579154. doi: 10.3389/fmicb.2025.1579154 (PMC12568640; doi:10.3389/fmicb.2025.1579154)
Supplement: Supplementary file 1 [file Data_Sheet_1.docx]

Virulence and mutations analysis based on the whole genome of a Brazilian *Corynebacterium diphtheriae* strain isolated from a cutaneous infection

Max Roberto Batista Araújo^1,2^**^†^**, Louisy Sanches dos Santos^3^**^†^**, Fernanda Diniz Prates^1,2^, Hugo Felix Perini^4^, Jailan Sousa Silva^2^, Juliana Nunes Ramos^3^, Sérgio Bokermann^5^, Cláudio Tavares Sacchi^6^, Ana Luiza de Mattos-Guaraldi^3^, Karoline Rodrigues Campos^6^, Tayná do Carmo Sant’Anna Cardoso^3^, Mireille Ângela Bernardes Sousa^1^, Verônica Viana Vieira^7^, Marlon Benedito Nascimento Santos^6^, Carlos Henrique Camargo^5^, Bruno Silva Andrade^8^, Marcos Vinicius da Silva^4^, Lincoln de Oliveira Sant’Anna^3^, Marcus Vinícius Canário Viana^2^, Vasco Azevedo^2*^

^1^ Operational Technical Nucleus, Microbiology, Hermes Pardini Institute (Fleury Group), Vespasiano, Minas Gerais, Brazil;

^2^ Department of Genetics, Ecology and Evolution, Federal University of Minas Gerais, Belo Horizonte, Minas Gerais, Brazil.

^3^ Laboratory of Diphtheria and Corynebacteria of Clinical Relevance, Department of Microbiology, Immunology and Parasitology, Rio de Janeiro State University, Rio de Janeiro, Rio de Janeiro, Brazil;

^4^ Institute of Biological and Natural Sciences, Federal University of Triângulo Mineiro, Uberaba, Minas Gerais, Brazil;

^5^ Center of Bacteriology, Adolfo Lutz Institute, Secretary of Health of the State of São Paulo, São Paulo, São Paulo, Brazil;

^6^ Strategic Laboratory, Adolfo Lutz Institute, Secretary of Health of the State of São Paulo, São Paulo, São Paulo, Brazil;

^7^ Interdisciplinary Laboratory of Medical Research, Oswaldo Cruz Institute, Oswaldo Cruz Foundation, Rio de Janeiro, Rio de Janeiro, Brazil;

^8^ Laboratory of Bioinformatics and Computational Chemistry, State University of Southwest Bahia, Jequié, Bahia, Brazil;

**^†^**These authors contributed equally to this work.

*Corresponding authors: Prof. Dr. Vasco Azevedo and Max Araújo. Adress: Departamento de Genética, Ecologia e Evolução, ICB/UFMG. Av. Antônio Carlos, 6627. Pampulha, Belo Horizonte, Minas Gerais, Brazil. CEP: 31270-901.

E-mails: vascoariston@gmail.com and max_barau@hotmail.com. Tel.: +55-31-3409-2610.

**Table S1.** Information about prophage regions found in the *Corynebacterium diphtheriae* 46855 isolate.

| **Region** | **Region Length** | **Completeness** | **Score** | **Total Proteins** | **Region Position** | **Most Common Phage** | **GC%** |
| --- | --- | --- | --- | --- | --- | --- | --- |
| 1  2  3 | 11Kb  17.1Kb  34.2Kb | Incomplete  Questionable  Questionable | 20  80  70 | 15  27  47 | (9) 25005 – 36026  (10) 23434 – 40541  (15) 8332 – 42545 | PHAGE_Bacill_G_NC_023719(1)  PHAGE_Gordon_GMA5_NC_030907(9)  PHAGE_Gordon_BritBrat_NC_030942(7) | 55.10%  55.67%  55.86% |

| **Table S2** – DDH *in silico* results obtained by GGDC calculator of *Corynebacterium diphtheriae* 46855 isolate in comparison with the most related taxa. | | | | | | | |  |
| --- | --- | --- | --- | --- | --- | --- | --- | --- |
| **Strain** | | ***Corynebacterium diphtheriae* NCTC11397^T^** | ***Corynebacterium belfantii***  **FRC0043^T^** | ***Corynebacterium***  ***rouxii***  **FRC0190^T^** | ***Corynebacterium pseudotuberculosis***  **ATCC 19410^T^** | ***Corynebacterium silvaticum***  **KL0182^T^** | ***Corynebacterium***  ***ulcerans***  **NCTC7910^T^** | ***Corynebacterium ramonnii* FRC0011^T^** |
| 46855 | 83.6% | | 61.6% | 48.7% | 21.3% | 21.7% | 21.8% | 21.5% |


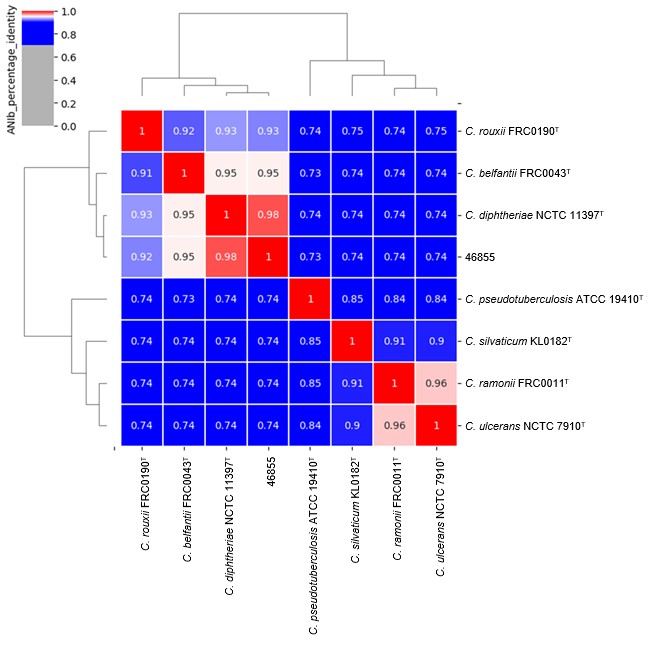
**Figure S1**. Heatmap of ANI values between 46855 strains and the outgroup genomes: *Corynebacterium rouxii* FRC0190 ^T^, *Corynebacterium belfantii* FRC0043^T^, *Corynebacterium diphtheriae* NCTC 11397^T^, *Corynebacterium pseudotuberculosis* ATCC 19410^T^, *Corynebacterium silvaticum* KL0182^T^, *Corynebacterium ulcerans* NCTC 7910^T^, and *Corynebacterium ramonii* FRC0011^T^.

**Figure S2**. The phylogenetic tree based on the sequences of the loci used in the MLST scheme of the *Corynebacterium diphtheriae* complex. The evolutionary model and phylogenetic inference were estimated by IQ-TREE2 program, with support values calculated by 1000 bootstrap replications.


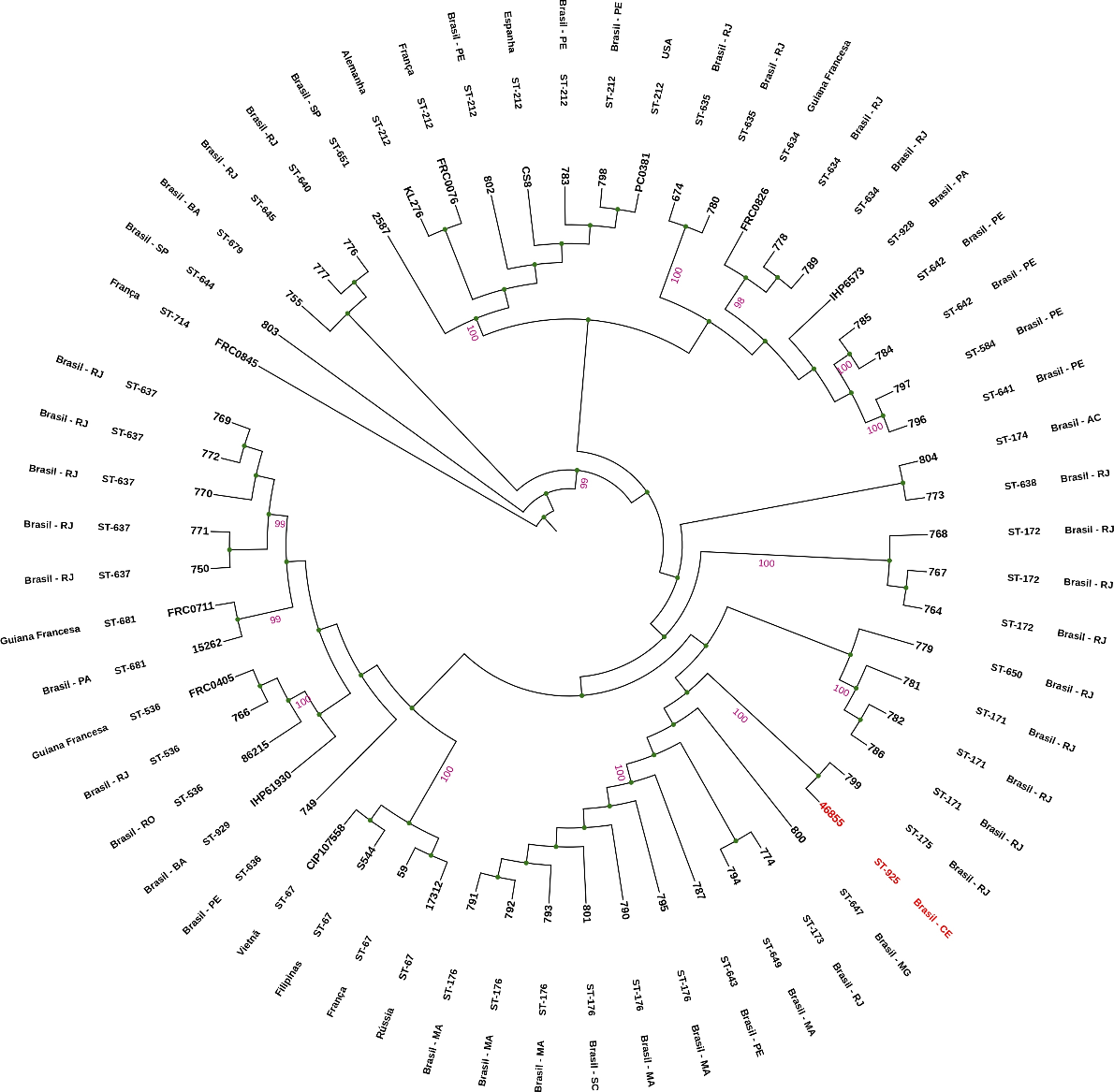


| **Table S3**. Virulence factors predicted in *Corynebacterium diphtheriae* 46855 isolate. | | | |
| --- | --- | --- | --- |
| **VF category** | | **Related genes** | **Product** |
| Adherence | SpaA-type pili | *spa*A | SpaA-type pili major subunit SpaA |
|  |  | *spa*B | SpaA-type pili minor subunit SpaB |
|  |  | *spaC* | SpaA-type pili minor subunit SpaC |
|  |  | *srt*A | fimbrial associated sortase |
|  | SpaD-type pili | *spa*D | SpaD-type pili major subunit SpaD |
|  |  | *spa*E | SpaE-type pili minor subunit SpaE |
|  |  | *spa*F | SpaA-type pili minor subunit SpaC |
|  |  | *srt*B | fimbrial associated sortase |
|  |  | *srt*C | fimbrial associated sortase |
|  | SpaH-type pili | *spa*G | SpaG-type pili major subunit SpaG |
|  |  | *spa*H | SpaH-type pili major subunit SpaH |
|  |  | *spa*I | SpaI-type pili major subunit SpaI |
|  |  | *srt*D | fimbrial associated sortase |
|  |  | *srt*E | fimbrial associated sortase |
|  | Surface-anchored pilus proteins | *sap*A | DUF11 domain-containing protein |
|  |  | *sap*D | choice-of-anchor M domain-containing |
|  |  | *sap*E | putative surface-anchored protein |
|  | Non-fimbrial adhesins | DIP_RS14950 | UPF0182 family protein - DIP0733 |
|  |  | DIP_RS19245 | C40 family peptidase - DIP1621 |
|  |  | DIP_RS17590 | C40 family peptidase - DIP1281 |
|  |  | *emb*C | arabinosyltransferase domain-containing protein |
|  | CdiLAM | *mpt*C | glycosyltransferase 87 family protein |
|  |  | *aft*B | arabinofuranosyl transferase C |
| Iron-uptake | ABC transporter | *fag*A | iron chelate uptake ABC transporter family permease subunit |
|  |  | *fag*B | iron chelate uptake ABC transporter family permease subunit |
|  |  | *fag*C | ABC transporter ATP-binding protein |
|  |  | *fag*D | iron-siderophore ABC transporter substrate-binding protein |
|  | ABC-type heme transporter | *hmu*T | ABC transporter substrate-binding protein |
|  |  | *hmu*U | iron ABC transporter permease |
|  |  | *hmu*V | heme ABC transporter ATP-binding protein |
|  |  | DIP_RS14425 | HtaA domain-containing protein - DIP0625 |
|  |  | DIP_RS14445 | HtaB domain-containing protein - DIP0629 |
|  |  | DIP_RS14420 | HtaC domain-containing protein - DIP0624 |
|  | Ciu iron uptake and siderophore biosynthesis system | *ciu*A | iron-siderophore ABC transporter substrate-binding protein |
|  |  | *ciu*B | iron ABC transporter permease |
|  |  | *ciu*C | iron ABC transporter permease |
|  |  | *ciu*D | Putative iron transport system ATP-binding protein |
|  |  | *ciu*E | siderophore biosynthesis protein |
|  | Siderophore-dependent iron uptake system | *irp6*A | ABC transporter substrate-binding protein |
|  |  | *irp6*B | iron ABC transporter permease |
|  |  | *irp6*C | ABC transporter ATP-binding protein |
| Transcription |  | *sig*A | RNA polymerase major sigma factor SigA |
| Regulation | Diphtheria toxin repressor | *dtx*R | MarR family transcriptional regulator |
| Post-translational modification | | DIP_RS20575 | thioredoxin domain-containing pr (MdbA) - DIP1880 |

| **Table S4.** Hits found to spacer sequences in the CRISPRTarget and CRISPR-Cas++ databases. | | | | | | | | |
| --- | --- | --- | --- | --- | --- | --- | --- | --- |
| **Isolate** | **Contig** | **Number of spacers** | **CRISPR-Cas++** | **Spacer** | **IC score** | **CRISPRTarget** | **Spacer** | **IC score** |
| **46855** | **9** | **4** | Cas-type I-E and II-C of *C. diphtheriae* 241 and HC01 | 1 | 1.00 | *C. diphtheriae* 241 and HC01 | 1 | 1.00 |
|  |  |  | Cas-type I-E and II-C of *C. diphtheriae* 241 and HC01 | 2 | 1.00 | *C. diphtheriae* 241 | 2 | 1.00 |
|  |  |  | Cas-type I-E and II-C of *C. diphtheriae* 241 and HC01 | 3 | 1.00 | *C. diphtheriae* 241 | 3 | 1.00 |
|  |  |  | Cas-type I-E and II-C of *C. diphtheriae* 241 and HC01 | 4 | 1.00 | *C. diphtheriae* HC01 | 4 | 1.00 |
|  | **3** | **5** | Cas-type I-E and II-C of *C. diphtheriae* HC01 | 2 | 0.96 | Phage Rhodoc RRH1 *C. diphtheriae* BH8 | 1 | 0.96 |
|  |  |  | Cas-type I-E and II-C of *C. diphtheriae* 241 and HC01 | 4 | 0.96 | *C. diphtheriae* B-D-16-78 Phage coryne BFK20 | 2 | 0.89 |
|  |  |  | Cas-type I-E and II-C of *C. diphtheriae* 241 and HC01 Cas-type II-C of *C. diphtheriae* 63e178e | 5 | 0.96 | Phage Rhodoc RRH1 (NC016800) *C. diphtheriae* NCTC13129 | 4 | 0.89 |
|  |  |  | - | - | - | *C. diphtheriae* PW8 *C. ulcerans* FRC58 Phage coryne BFK20 | 5 | 1.00 |

**Figure S3.** Interactions between Y441 and F445 with partial conservation of conserved contacts**.** The residues present in the wild-type version of the protein are in yellow, while those in the mutated version are in purple. A) Match between Asn_441 and Tyr_441 in the protein's three-dimensional space; however, in both cases, the contacts that used to be hydrogen bonds now have a hydrophobic interaction.

**
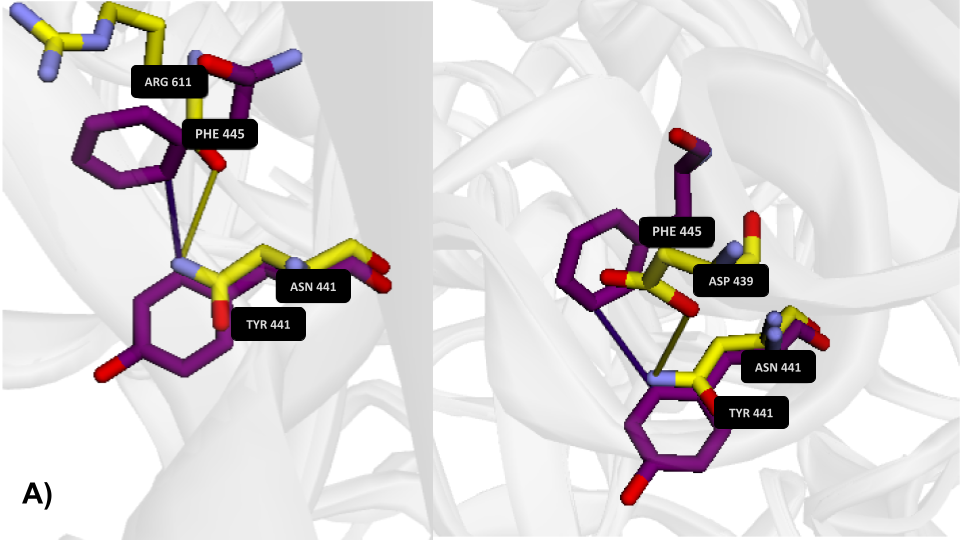
**

**Table S5**. Interactions carried out by each amino acid residue present in the positions of interest according to DynaMut2.

| **Residue/Interactions** | **Clash** | **Ionic** | **Carbonyl** | **VDW** | **Aromatic** | **Polar** | **Hydrogen bond** | **Hydrophobic** |
| --- | --- | --- | --- | --- | --- | --- | --- | --- |
| **Asparagine/Asp/N at position 441 (WT)*** | *His_1033 (2), Gln_678, Arg_611, Pro_615 (2) | - | - | Ser_445, Asp_439, Gln_678 | - | Gln_678 (2), His_1033 (2), Arg_611 (2), Pro_615 (2), Asp_439, Ser_445. | Gln_678, Pro_615, Arg_611, Asp_439, Ser_445 | - |
| **Serine/Ser/S at position 445 (WT)** | Asp_439, Asn_442 (3) | - | Asp_439 | Asp_439, His_449, Thr_448, Asn_441 | - | Asp_442 (7), Asp_439, His_449 (2), Thr_448 | Asp_441, Asn_442 (5), Arg_611, Thr_448 (2),  His_449 | - |
| **Tyrosine/Tyr/Y at position 441 (M)*** | Pro_615, His_1033 (2), Gln_678 | - | - | Gln_678 (2) | Phe_445 (3) | His_1033, Ala_613, Thr_680, Pro_615,  Arg_611 | Pro_615, Ala_613, Arg_611 (2) | His_1033 (5), Gln_678 (4), Thr_680, Phe_445 (5) |
| **Phenylalanine/Phe/F at position 445 (M)** | Thr_448, His_449, Asp_439, Arg_611 (4),  Asn_442 | - | - | Arg_611 (3), Asp_439, Leu_447 | Tyr_441 (3),  His_1033 (9) | Arg_611 (3), Asn_442 (3), Ser_443, Leu_447, Thr_448 (2),  His_449 (2),  Asp_439. | Asn_442 (3), Thr_448 (3), | Asp_439 (6),  Tyr_441 (5), Arg_611 (10) |

***Amino acid residue, followed by position and number of interactions in brackets. *WT = Wild Type version. *M = Mutated version.**

**Table S6**. Docking scores and rifampin interactions type with binding site amino acids.

| 5UHC_B | | *rpoB-*N441Y-S445F | |
| --- | --- | --- | --- |
| Affinity(Kcal/mol) | -10.1 | -7.7 | |
| Binding site amino acids | interactions | Binding site amino acids | interactions |
| PHE-439 | HB | PHE-437 | Pi-Alkyl |
| SER-434 | HB | _ | _ |
| GLN-435 | HB | GLN-433 | HB |
| LEU-436 | Alkyl | _ | _ |
| ASN-443 | _ | TYR-441 | Pi-sigma |
| ARG-454 | HB | _ | _ |
| LEU-458 | Pi-Alkyl/Alkyl | _ | _ |
| ARG-465 | Repulsive | ARG-463 | HB/Repulsive |
| PRO-489 | Alkyl | PRO-487 | Alkyl |
| ILE-497 | Pi-Alkyl | _ | _ |
| HIS-680 | Alkyl | _ | _ |

**Figure S4**. Superposition of 5HUC_B (blue) and rpoB*-*N441Y-S445F (orange) in rifampin binding site at 5 angstroms contacts, highlighting amino acid substitutions.


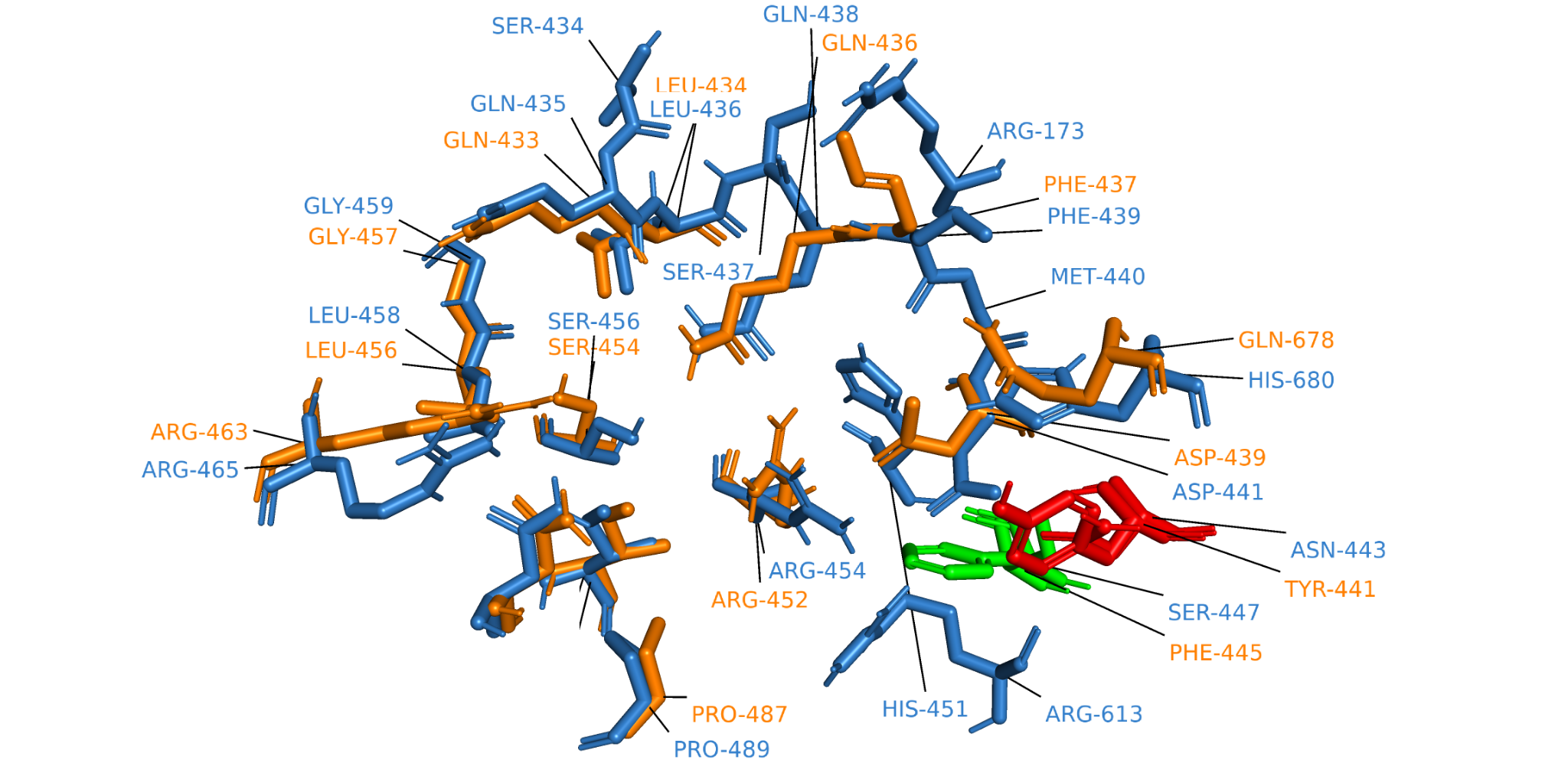


**Figure S5**. Root mean square deviation of rifampin after last square fit to rpoB*-*N441Y-S445F for one hundred nanoseconds simulation time.

**
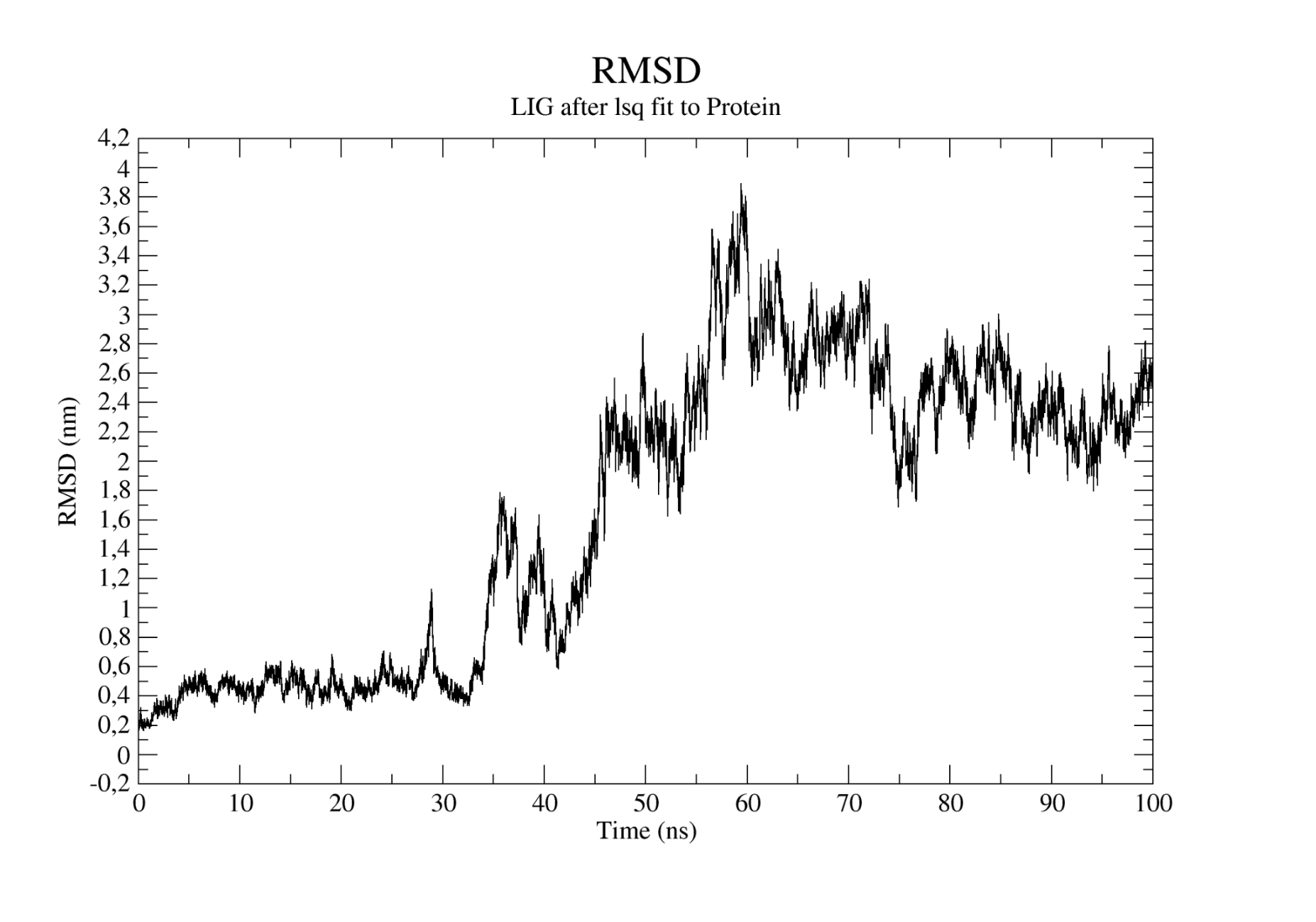
**
